# Weakening Impact of Excessive Human Serum Albumin (eHSA) on Cisplatin and Etoposide Anticancer Effect in C57BL/6 Mice with Tumor and in Human NSCLC A549 Cells

**DOI:** 10.3389/fphar.2016.00434

**Published:** 2016-11-15

**Authors:** Zhen Yang, Ting Zhou, Yuanchi Cheng, Mingming Li, Xianglin Tan, Feng Xu

**Affiliations:** ^1^Fengxian Hospital Graduate Training Base, Jinzhou Medical UniversityShanghai, China; ^2^Graduate School, Jinzhou Medical UniversityLiaoning, China; ^3^Department of Pharmacy, Fengxian Hospital, Southern Medical UniversityShanghai, China; ^4^Rutgers Cancer Institute of New Jersey, The State University of New JerseyNew Brunswick, NJ, USA; ^5^Department of Pharmacy, 6th People’s Hospital South Campus, Shanghai Jiaotong UniversityShanghai, China

**Keywords:** human serum albumin, cisplatin, etoposide, A549, C57BL/6 mice

## Abstract

Excessive human serum albumin (eHSA) impact on anticancer effects is inconsistent. We explored the outcome of cisplatin (DDP)/etoposide (VP-16) plus eHSA *in vivo* and *in vitro*. C57BL/6 mice with tumor were used to compare the efficacy of DDP/VP-16 alone and DDP/VP-16+eHSA. Blood albumin was measured to confirm whether eHSA elevate its level. Western blotting assay were used to measure the expression of ERCC1/TOP2A in tumor tissues. Cell proliferation, mRNA, and protein expression of ERCC1/TOP2A were also assayed to compare two groups in A549 cells. Furthermore we evaluated eHSA impact on cell proliferation in RNAi targeting ERCC1/TOP2A in A549 cells, respectively. eHSA reduced the anticancer effect of DDP/VP-16 without altering albumin level, increased protein expression of ERCC1/TOP2A, respectively in mice. Similarly, eHSA increased mRNA and proteins expression of ERCC1/TOP2A in A549 cells. In RNAi A549 cells, however, eHSA no longer weakened but enhanced the anticancer effect of DDP, while no longer altered the effect of VP-16. Our findings suggested that eHSA weaken the anticancer effect of DDP/VP-16 via up-regulating ERCC1/TOP2A expression, respectively. Further molecular mechanism studies are warranted to investigate whether eHSA is not conducive to lung cancer chemotherapy.

## Introduction

Human serum albumin (HSA) has been used in clinical practice for more than 60 years, which is mainly used for resuscitation and volume expansion in critically ill patients (Liberati et al., 2006). It is the most abundant protein in plasma and one of the major binders/carriers of drugs that plays an important role in pharmacokinetic fate (absorption, distribution, metabolism, and excretion) and pharmacodynamics (Kratz and Beyer, 1998; Leonis et al., 2015; Alam et al., 2016; Ma et al., 2016; Zaloga et al., 2016). Since the half-life of HSA is about 15–21 days, it cannot be immediately utilized by the human body, which means that exogenous HSA cannot correct hypoproteinemia immediately in cancer patients (Sikuler et al., 2000; Talasaz et al., 2012).

The administration of HSA has been the subject of intense debate in China, because HSA is usually abused as tonic for cancer patients, the older and any other frail people. A few *in vitro* studies results of the excessive human serum albumin (eHSA) supplement impact on chemotherapy is inconsistent (Takahashi et al., 1980).

Lung cancer is the most common malignant tumors which is a leading cause of cancer mortality in China (Siegel et al., 2013), and the routine post-operation chemotherapy is cisplatin (DDP)/etoposide (VP-16) regimen plus HSA supplement in China. According to the *USA HSA Clinical Application Guide*, the indication of HSA is limited within shock, burns, ARDS, cardiopulmonary bypass, acute liver failure, and kidney dialysis occasionally. When HSA level ranges from 15 to 20 g/L, whether HSA use or not is decided by the specific circumstances of the patient. However, many lung cancer patients who neither appear hypoproteinemia nor have any indications for HSA supplement were still prescribed with HSA. eHSA supplement might cause many adverse reactions including anaphylaxis, drug fever, kidney damage, heart failure, hemolysis, pulmonary edema, and other serious incidents (Zhou et al., 2013). Whether eHSA supplement effect the chemotherapy is worth exploring.

In this study, we hypothesized that eHSA may impact anticancer effect *in vivo* and *in vitro*. We quantitatively detected the anticancer effect of DDP/VP-16, mRNA and protein expression of ERCC1/TOP2A in C57BL/6 mice with tumor and in human NSCLC A549 cells, as well as in RNAi A549 cells. Meanwhile, we evaluated the effects of eHSA supplement on blood albumin level in mice with tumor.

## Materials and Methods

### Materials

Cisplatin (DDP) was obtained from Qilu Pharmaceuticals, Co. Ltd (Jinan, China). Etoposide (VP-16) was obtained from Jiangsu Hengrui Pharmaceutical, Co. Ltd (Jiangsu, China). HSA (powder) was purchased from Sigma (St. Louis, MO, USA). HSA was purchased from CSL Behring AG (CH). All materials for cell culture were purchased from Gibco (Life Technologies, USA). Cholecystokinin octapeptide (CCK-8) was purchased from Ruian Biological, Co. (Shanghai, China). Antibodies against topoisomerase 2α and ERCC1 were obtained from Abcam (USA). The second antibody goat anti-rabbit IgG were purchased from Merck. Tubulin and GAPDH anti-rabbit and Beyo ECL Star were obtained from Beyotime Biological, Co. (Shanghai, China). SiRNA-mate was purchased from Shanghai Gene Pharma, Co., Ltd (Shanghai, China). TliRNaseH Plus was purchased from Takara Bio Inc (RR420A, Japan). BCA Protein Assay Kit was purchased from Thermo scientific (Waltham, MA, USA). PVDF membrane was purchased from Millipore Corp (Bedford, MA, USA). Chemiluminescence (ECL) detection kit was purchased from Thermo Fisher (Rockford, IL, USA).

### Cell Culture and Animals

Human NSCLC cell line A549 and mice NSCLC cell line LLC was obtained from Shanghai Institutes of Cell Biology (Shanghai, China). Cells were cultured in DMEM medium, supplemented with 10% fetal bovine serum (FBS), 100 μg/mL streptomycin, 100 units/ml penicillin at 5% CO_2_ at 37°C.

C57BL/6 male mice of 8-week-old (16–20 g) were purchased from Academy of Military Medical Sciences (Beijing, China). Animal (batch number: 1402491) were kept in the Laboratory Animal Center, East China Normal University, Shanghai (Animal Experiment License: SYXK 2010-0094). This study was carried out in accordance with the recommendations of Institutional Ethics Committee. Normal mice were set as control without any treatment. All mice inoculated with tumor cells were given 1, 2, 4 g/kg/d of HSA i.v. via tail vein for 3 days respectively, and then were randomized into 12 groups as follows: Group A: NS + NS, Group B: HSA (1 g/kg/d) + NS, Group C: HSA (2 g/kg/d) + NS, Group D: HSA (4 g/kg/d) + NS, Group E: NS + DDP, Group F: HSA (1 g/kg/d) + DDP, Group G: HSA (2 g/kg/d) + DDP, Group H: HSA (4 g/kg/d) + DDP, Group I: NS + VP-16, Group J: HSA (1 g/kg/d) + VP-16, Group K: HSA (2 g/kg/d) + VP-16, Group L: HSA (4 g/kg/d) + VP-16. On day 4 the mice were given 2, 10 mg/kg/d of DDP/VP-16 i.p. for 18 days, respectively.

### Detection of Blood Albumin Level, Body Weight, and Tumor Inhibition Rate of Mice

LLC lung cancer cells (1 × 10^7^ cells) suspended in 0.2 mL PBS was subcutaneously inoculated in the right flank of C57BL/6 mice. The model was established at day 10 after inoculation. After HSA administration 3 days later, the blood albumin level was detected before the chemotherapy initiation (on day 4) and at the end of treatment (on day 21). Blood albumin level was detected by using Beckman Coulter (AU5800). During the treatment, tumor length (L) and width (W) was calibrated using caliper twice a week and tumor volume was calculated as the formula: V = L × W^2^/2. After treatment, tumors were isolated and weighed. The inhibition rate was calculated as ratio of (1-tumor weight of experiment group/control group) × 100%.

### Western Blotting Assay for ERCC1, TOP2A Proteins Expression

After being treated with indicated drugs, tumor tissue (groups A, C, E, G, I, K) were sliced and sonicated while cells were collected in lysis buffer on ice. The protein was quantified with BCA protein assay kit. Total protein (25 μg) from each sample were run on SDS-PAGE and electrophoretically blotted onto PVDF membrane. The blots were blocked with 5% fat-free milk in Tris-buffered saline-Tween 20 (TBST) at room temperature for 4 h, followed by incubated with antibody at 4°C overnight. All antibodies were diluted with 5% fat-free milk in TBST buffer according to the instructions. The blots were washed with TBST buffer three times (10 min each time) at room temperature, and then labeled with secondary antibody at room temperature for 2 h respectively. Protein bands were detected using an enhanced chemiluminescence (ECL) detection kit (He et al., 2015).

### Cell Viability Assay by CCK-8

Cells (5000 cells per well) were plated into 96-well plates, then treated with DDP, VP-16, HSA alone or DDP+HSA, VP-16+HSA for 72 h. Cell viability was assayed using CCK-8 according to the manufacturer’s instructions. The absorbance of OD at 450 nm was detected and recorded with GloMax-Muiti+(Promega, E8032, USA).

### Determination of mRNA Expression

Cells were seeded into 6-well plates and treated as above for 72 h. Total RNA was extracted with Trizol according to the protocol (Sangon Biotech, SK1312/BS409, Shanghai, China) and RNA concentration were measured with NanoDrop ND-100 Spectrophotometer (Thermo Scientific, Wilmington, DE, USA). For qRT-PCR, TliRNaseH Plus was used according to the manufacture’s protocol (Sun et al., 2015). The primers sequences are as follows: ERCC1, forward 5′-CATCGCCGCATCAAGAGA-3′, reverse 5′-TTGGGGTCTCAGGTTGTGTTT-3′; TOP2A, forward 5′-CAAACTCGATGCCAATGA-3′, reverse 5′-GTCTCTCCCAACCACACCAAG-3′; GAPDH, forward 5′-GTCTTCACCACCATGGAGAAGG-3′, reverse 5′-GGCAGGTCAGGTCCACTGA-3′.

### RNA Interference (RNAi)

A549 Cells (150000 cells per well) were seeded into 6-well plates for 24 h, ERCC1/TOP2A were respectively interfered with OPTI-MEM 500 μL +siRNA-mate 1000 pmol +corresponding siRNA 1000 pmol. Then cells treated with DDP, VP-16, HSA alone or DDP+HSA, VP-16+HSA combination for 72 h and collected for Western blotting and qRT-PCR.

A549 cells (5000 cells per well) were seeded into 96-well plates for 24 h, ERCC1 and TOP2A were respectively interfered with OPTI-MEM 50 μL+siRNA-mate 100 pmol+ corresponding siRNA 80 pmol. Eight hours later, cells treated with DDP, VP-16, HSA alone or DDP+HSA, VP-16+HSA combination for 72 h and collected for CCK-8 test.

The primers of corresponding siRNA are as follows: ERCC1-homo-294 sense 5′–3′ GCCAAGCCCUUAUUCCGAUTT, antisense 5′–3′ AUCGGAAUAAGGGCUUGGCTT; TOP2A-homo-3522 sense 5′–3′ GACCAACCUUCAACUAUCUTT, antisense 5′–3′ AGAUAGUUGAAGGUUGGUCTT.

### Statistical Analysis

Data were expressed as mean ± SD. Statistical comparisons between the two groups were performed using the Student’s *t*-test and the intergroup were performed using the One-way analysis of variance (ANOVA). All analyses were performed using the statistical package for the social sciences (SPSS) 19.0, and (two-tailed) *P* < 0.05 was considered to be statistically significant.

## Results

### Impact of eHSA Supplement on Blood Albumin Level and DDP/VP-16 Anticancer Effects in Mice with Tumor

To evaluate the effects of eHSA supplement on DDP/VP-16 anticancer effects in mice with tumor, we performed *in vivo* assay in C57BL/6 mice. Normal mice were set as control without any treatment. All mice inoculated with tumor cells were given HSA for 3 days, and then were randomized into 12 groups as follows: Group A: normal saline (NS) + NS, Group B: HSA (L) (low dose of HSA) +NS, Group C: HSA (M) (medium dose of HSA) +NS, Group D: HSA (H) (high dose of HSA) +NS, Group E: NS+DDP, Group F: HSA (L) +DDP, Group G: HSA (M) +DDP, Group H: HSA (H) +DDP, Group I: NS+VP-16, Group J: HSA (L) +VP-16, Group K: HSA (M) +VP-16, Group L: HSA (H) +VP-16. DDP/VP-16 was given respectively for 18 days. Blood albumin level was detected before the chemotherapy initiation (day 4th) and at the end of treatment (day 21st).

**FIGURE 1:**
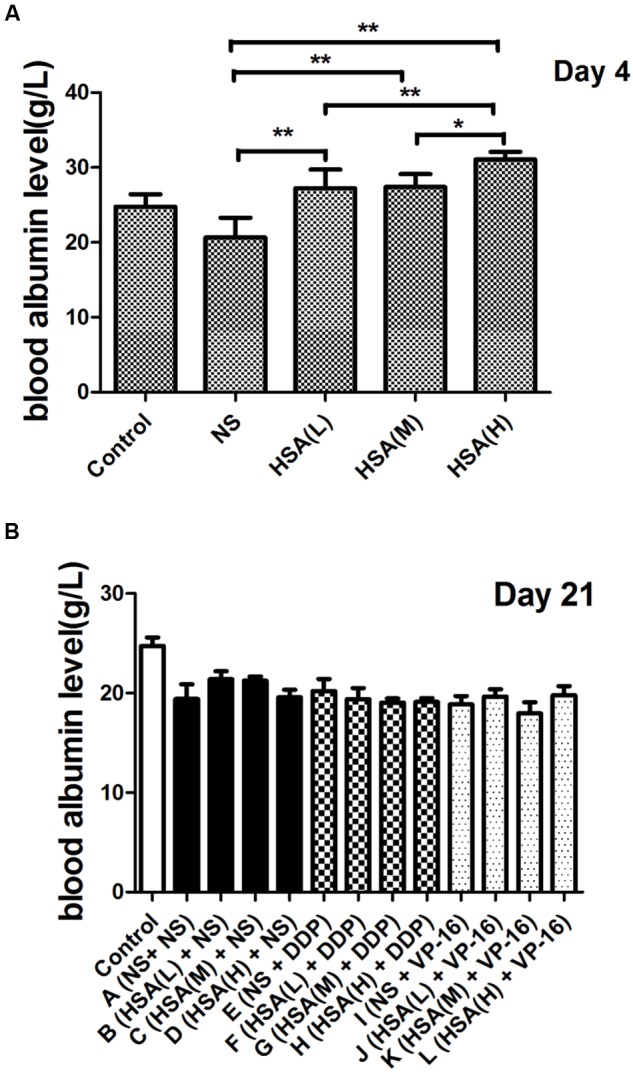
**Blood albumin levels of each group at various time points. (A)** Comparison of blood albumin levels between different groups at the end of different concentrations of HSA treatment. **(B)** Comparison of blood albumin levels between different groups at the end of anticancer drugs treatment. ^∗^*P* < 0.05, ^∗∗^*P* < 0.01.

**FIGURE 2:**
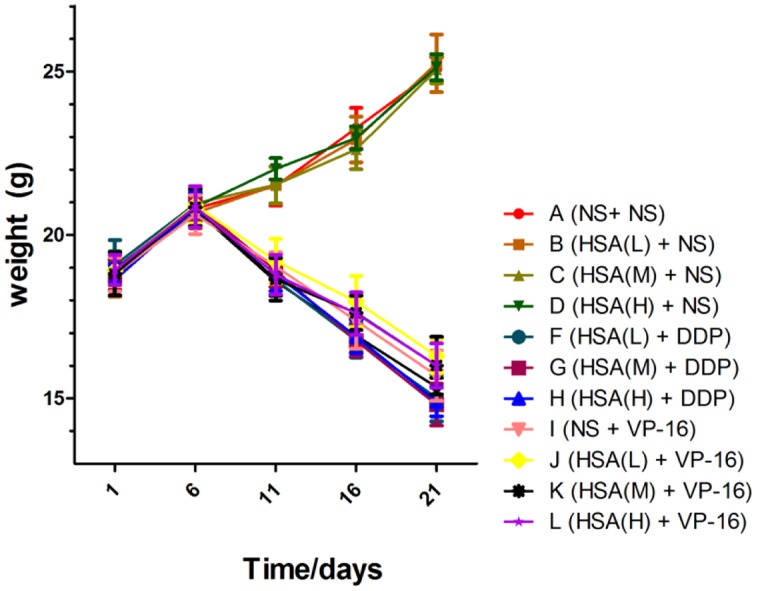
**Body weight of each group at various time points.** Time starting from HSA treatment.

The results displayed that blood albumin levels were positively correlated with HSA supplement in mice before the chemotherapy initiation [HSA (H) > HSA (M) > HSA (L) > NS]. However, at the end of treatment their levels in 12 groups were almost the same, suggesting that supplemented HSA was catabolized completely with the time (**Figure 1**). At the end of treatment, the body weight of mice in Groups A ∼ D increased gradually, meanwhile decreased gradually in Groups E ∼ H and Groups I ∼ L. However, the body weight of mice in Group E was slightly less than that Groups F, G, and H. Similarly, the body weight of mice in Group I was slightly less than that in Groups J, K, and L, although no statistically difference existed among these groups (*P* > 0.05) (**Figure 2**).

**FIGURE 3:**
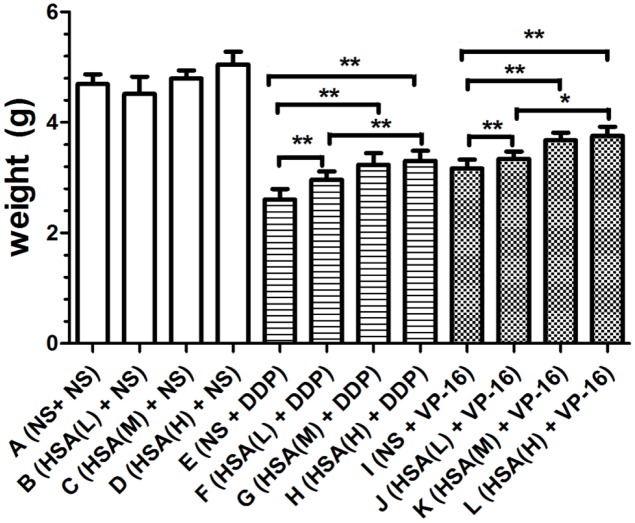
**Inoculated tumor weight of each group at the end of treatment.** ^∗^*P* < 0.05, ^∗∗^*P* < 0.01.

**FIGURE 4:**
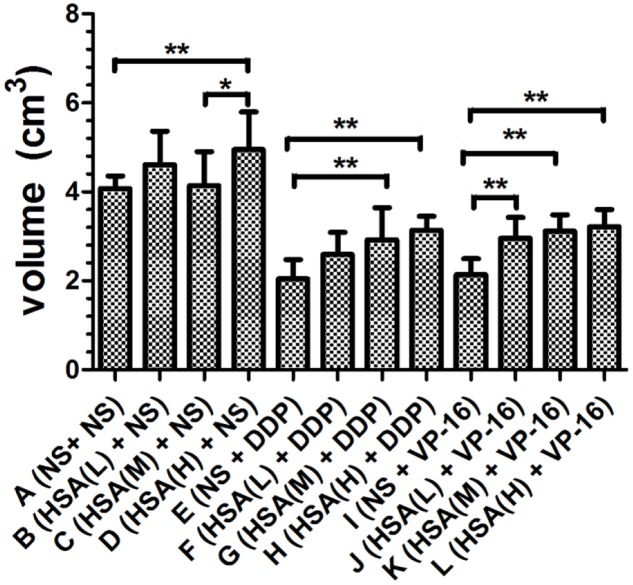
**Volume of inoculated tumor of each group at the end of treatment.** ^∗^*P* < 0.05, ^∗∗^*P* < 0.01.

Furthermore, we observed that the tumor weight in Groups E and I decreased more than that in Groups F, G, H and Groups J, K, L, respectively (*P* < 0.05) (**Figure 3**). The tumor growth inhibition rates of DDP treatment Groups E, F, G, H were 44.35, 36.89, 30.92, 29.42%, respectively. The tumor growth inhibition rates of VP-16 treatment Groups I, J, K, L is 32.41, 28.78, 21.54, 19.83%, respectively. Similarly, the tumor volume in Groups E and I decreased more than that in Groups F, G, H and Groups J, K, L, respectively (*P* < 0.05) (**Figure 4**). These results suggested that eHSA supplement weakened the anticancer effects of DDP and VP-16 in mice with tumor.

**FIGURE 5:**
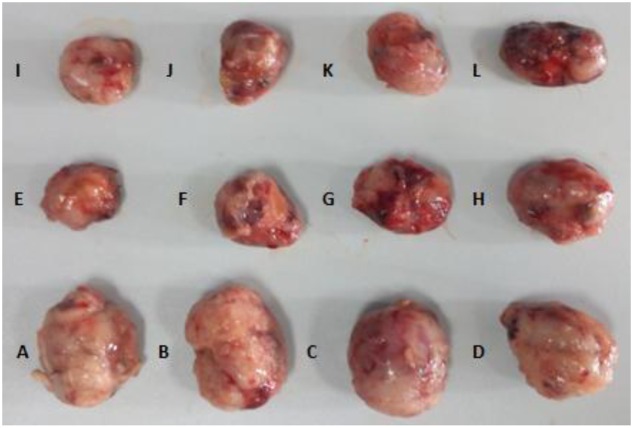
**Tumor tissue of each group at the end of treatment. (A)** NS+ NS, **(B)** HSA (L) + NS, **(C)** HSA (M) + NS, **(D)** HSA (H) + NS, **(E)** NS + DDP, **(F)** HSA (L) + DDP, **(G)** HSA (M) + DDP, **(H)** HSA (H) + DDP, **(I)** NS + VP-16, **(J)** HSA (L) + VP-16, **(K)** HSA (M) + VP-16, **(L)** HSA (H) + VP-16.

Histopathological results displayed that the tumor tissue was soft with smooth or festering surface in Groups A, B, C, and D. In Groups E ∼ L, the tumor texture was very hard with a slightly uneven surface, more bleeding as compared with that in Groups A ∼ D (**Figure 5**).

### Impact of eHSA on Protein Expression ERCC1 and TOP2A in Tumor Tissue

**FIGURE 6:**
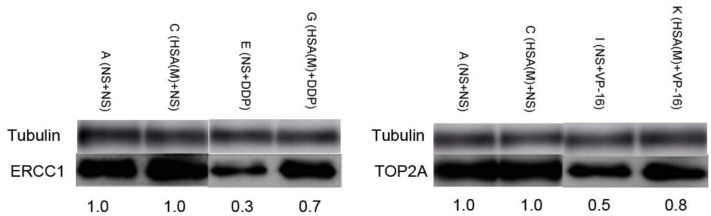
**Impact of eHSA on protein expression of ERCC1 and TOP2A in tumor tissues.** HSA concentration is 2 g/kg/d, all samples were collected at the end of treatment.

ERCC1 and TOP2A are prominent targets of DDP and VP-16, respectively (Verdier-Pinard et al., 2003; Olaussen et al., 2006). We further investigated whether eHSA weakend the anticancer effeect via regulating ERCC1 and TOP2A expression in mice. At the end of treatment, tumor tissue in mice of Groups A, C, E, G, I, K was isolated and proteins were detected by Western blotting. We found that eHSA supplement significantly enhanced protein expression of ERCC1 and TOP2A compared with DDP/VP-16 monotherapy group (**Figure 6**).

### Impact of eHSA Supplement on DDP/VP-16 Anticancer Effect in A549 Cells and RNAi A549 Cells

**FIGURE 7:**
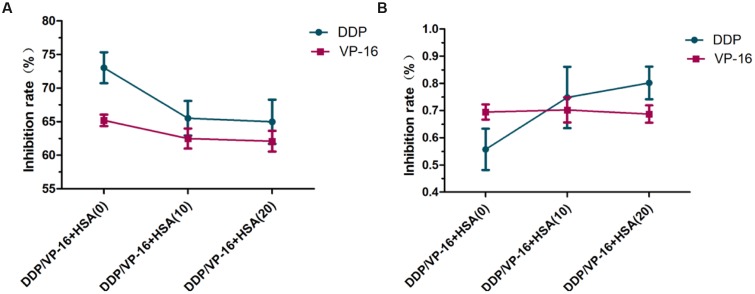
**Effect of HSA on inhibition rate of DDP and VP-16 in A549 cells and RNAi A549 cells. (A)** Effect of HSA on inhibition rate of DDP and VP-16 in A549 cells. **(B)** Effect of HSA on inhibition rate of DDP and VP-16 in RNAi A549 cells. Date are mean ± SD, *n* = 3 means of triplicate measures.

**FIGURE 8:**
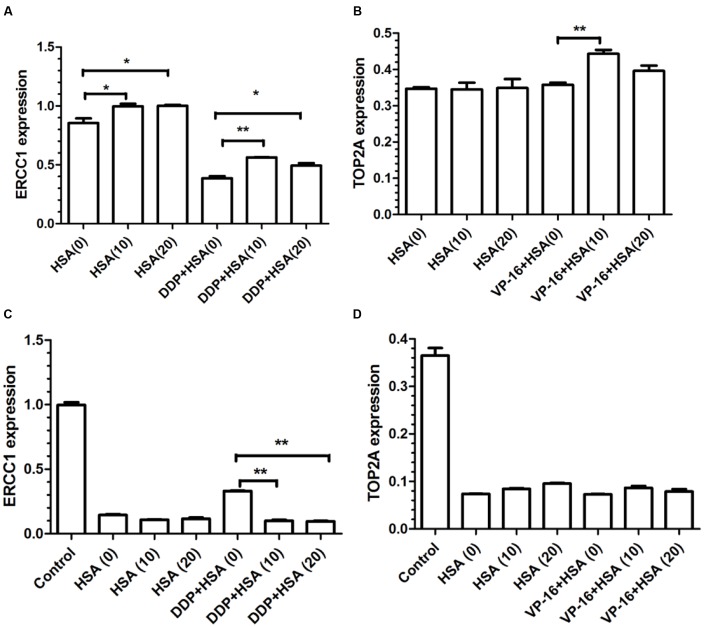
**Effects of HSA on mRNA expression of ERCC1 and TOP2A in A549 cells and RNAi A549 cells. (A,B)** The mRNA expression of ERCC1 and TOP2A from statistical analysis of three independent experiments in A549 cells. **(C,D)** The mRNA expression of ERCC1 and TOP2A from statistical analysis of three independent experiments in RNAi A549 cells. ^∗^*P* < 0.05, ^∗∗^*P* < 0.01.

**FIGURE 9:**
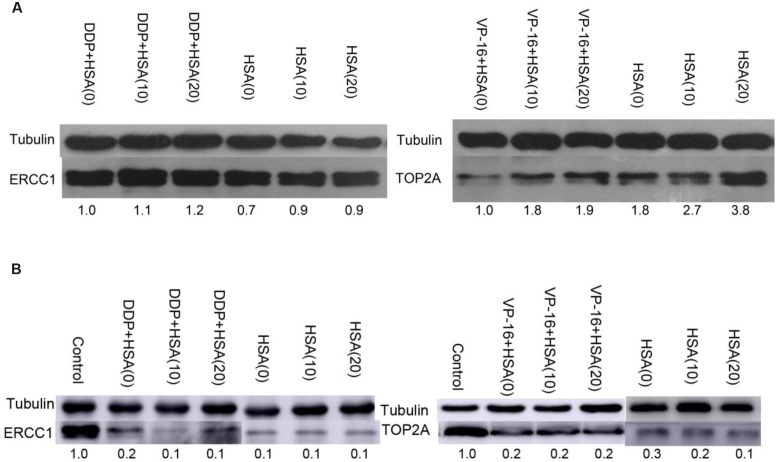
**Effects of HSA on protein expression of ERCC1 and TOP2A in A549 cells and RNAi A549 cells. (A)** The protein expression of ERCC1, TOP2A was detected by Western blotting in A549 cells. **(B)** The protein expression of ERCC1, TOP2A was detected by Western blotting in RNAi A549 cells.

To verify eHSA weakening anticancer effect *in vitro*, we evaluated the impact of eHSA on cell viability with/without DDP/VP-16 treatment in human NSCLC A549 cells and RNAi A549 cells. We performed CCK-8 assay by exposure of the cells to 10 μmol/L DDP/VP-16 and 10/20 μmol/L HSA for 72 h. The results showed that eHSA significantly weakened the inhibiting effect of DDP/VP-16 in A549 cells (**Figure 7A**). However, in RNAi A549 cells, eHSA no longer weakened but enhanced the inhibiting effect of DDP alone, meanwhile eHSA no longer altered the effect of VP-16 alone (**Figure 7B**). Similarly, eHSA supplement significantly enhanced the mRNA expression of ERCC1 and TOP2A compared with the monotherapy group in A549 cells (**Figures 8A,B**). In ERCC1 and TOP2A RNA interfered A549 cells, mRNA expression of ERCC1 and TOP2A were apparently reduced (**Figures 8C,D**). eHSA supplement enhanced the protein expression of ERCC1 and TOP2A compared with the monotherapy group in A549 cells (**Figure 9A**). In ERCC1 and TOP2A RNA interfered A549 cells, protein expression of ERCC1 and TOP2A were significantly decreased (**Figure 9B**). These results suggested that ERCC1 and TOP2A might be involved in cell viability in A549 cells.

## Discussion

The administration of HSA is a controversial topic in clinical practice especially in China, whether the use HSA is beneficial or not is unclear. Although, the indication of HSA is limited according to the *USA HSA Clinical Application Guide*, it has been abused as a tonic both in developed areas and in remote backward areas in China. It is often inadvisable recommended for serious diseases such as malignant tumors by family members (Grootendorst et al., 1988; Finfer et al., 2004; Morris et al., 2014). In clinical practice HSA is only recommended to use for critical situation as HSA level is less than 15 g/L. Our preliminary survey in lung cancer patients displayed that about 30% of patients with HSA level larger than 35 g/L were still prescribed with HSA supplement. However, up to now, the impact of eHSA on efficacy of chemotherapy is not clear.

Takahashi et al. (1980) reported the impact of HSA on 13 anticancer agents in human leukemia cell MOLT-3. Three outcomes of anti-cancer activity were observed: decreased, unchanged, and enhanced (Takahashi et al., 1980). Our work focused on the anticancer effect of DDP, VP-16 in combination with eHSA *in vivo* and *in vitro*. We found that eHSA supplement reduced the anticancer effect of DDP/VP-16 without altering blood albumin level as compared with the corresponding monotherapy group in mice with tumor. Excessive HSA significantly up-regulated the protein expression of ERCC1 and TOP2A respectively in mice with tumor. *In vitro*, eHSA weakened the anticancer effect of DDP/VP-16 in a dose-dependent manner by up-regulating the protein and mRNA expression of ERCC1/TOP2A in A549 cells. Remarkably, we showed that in ERCC1/TOP2A RNAi A549 cells, eHSA no longer weakened but enhanced the anticancer effect of DDP, while no longer altered the effect of VP-16, respectively.

The *in vivo* results demonstrated that eHSA supplement did not affect the body weight of mice. The blood albumin level was positively related to the HSA dose on day 4. Interestingly, HSA levels in 12 groups were almost the same at the end of treatment, suggesting that supplemented HSA was catabolized completely with the time (Powell et al., 2013).

ERCC1/TOP2A is resistance gene of DDP/VP-16, respectively (Verdier-Pinard et al., 2003; Olaussen et al., 2006). Our results showed that eHSA enhanced mRNA/protein expression of ERCC1 and TOP2A both *in vivo* and *in vitro*. However, in ERCC1 RNAi and TOP2A RNAi A549 cells, eHSA increased the effect of DDP while no longer altered the effect of VP-16. These data suggested that ERCC1 might be one of targets by which eHSA weakened the anticancer effect of DDP. We suspect there are another pathway regulate efficacy of DDP which altered by HSA (Chen et al., 2016). eHSA might weaken anticancer effect of VP-16 via up-regualting TOP2A exclusively. Based on these evidences above, we reason that eHSA supplement may reduce the clinical efficacy of anticancer drugs. Excessive HSA supplement might not benefit for lung cancer patients with normal albumin levels (Song et al., 2011).

Different HSA-drug interaction outcomes (Takahashi et al., 1980) suggested that the impact of HSA supplement (especially eHSA supplement in China) might be drug-specific, disease-specific which cannot be generalized (Tang et al., 2011; Punith and Seetharamappa, 2012; Yousefi et al., 2012; Wu et al., 2013). It is of importance to individualize HSA use for cancer patients. Further *in vivo* experiments and mechanism studies are warranted to investigate whether eHSA exhibits is not conducive to therapy of lung cancer patients.

## Conclusion

In summary, our research about eHSA combination with DDP/VP-16 *in vivo* and *in vitro* showed negative effect to anticancer drugs, which suggested that eHSA weaken the anticancer effect of DDP/VP-16 via up-regulating ERCC1/TOP2A expression, respectively. But further molecular mechanism studies are warranted to investigate whether eHSA is not conducive to lung cancer chemotherapy.

## Author Contributions

Conceived and designed the experiments: TZ, XT, and FX. Performed the experiments: ZY. Analyzed the data: ZY. Contributed reagents/materials/analysis tools: ZY, FX, YC, and ML. Wrote the paper: ZY. Revised the manuscript: FX. Contributed to data collection: TZ, ZY, YC, ML, XT.

## Conflict of Interest Statement

The authors declare that the research was conducted in the absence of any commercial or financial relationships that could be construed as a potential conflict of interest.
